# The Yellow-Fever Question

**Published:** 1900-11-17

**Authors:** 


### THE YELLOW-FEVER QUESTION.

The present position of the yellow-fever question is a matter of much interest, especially in view of recent statements as to the part played by the mosquito in its transmission from man to man. As is well known, yellow fever can only be contracted within the area where it is endemic, the infection appearing to be derived from the house, the ship, or the locality in which previous cases have occurred rather than directly from these patients themselves. Again, it has been pointed out that after the introduction of the infection into an unaffected locality some little time must elapse before this place becomes in turn an endemic centre of the disease. In other words, a longer time than the ordinary period of incubation is found to pass by after the arrival of a patient suffering from the disease before secondary cases occur. It seems not unreasonable to believe that during this time the infective organism—whatever its nature, whether microphytic or protozoal—passes through some stage of its existence outside the human body; and if it should turn out, as has been so long suspected, that it is by the mosquito that the infection is implanted upon man, analogy with malaria would very strongly suggest that it may be within the body of the mosquito that this further stage is passed.

A somewhat rough-and-ready experiment has lately been tried in Cuba. Seven persons who were believed not to be immune, were subjected to the bites of mosquitoes which had been fed from two to eight days previously on patients suffering from various stages of yellow fever. The result was nil; none of the persons bitten were any the worse for it. This might at first sight be taken as negativing the mosquito theory. But no. By lengthening the interval between the feeding of the mosquitoes and the infection of the non-immune person, that is, by allowing ten to thirteen days instead or from two to eight days, to elapse, between the feeding and the biting, a different result was obtained, two out of the four persons thus bitten having developed undoubted yellow fever. There are many difficulties and many chances of error in experiments of this kind, when conducted in localities in which the disease in question is endemic; but if a repetition of similar experiments, under more rigid conditions, should give the same results, there would certainly be strong grounds for believing that one more disease is to be added to the list of those which man owes to that peculiarly objectionable creature, the mosquito.

